# A newly identified sequence type: genomic and pathogenic profiling of the highly virulent *Acinetobacter baumannii* ST3475 from bovine mastitis

**DOI:** 10.3389/fcimb.2026.1804085

**Published:** 2026-04-14

**Authors:** Tian Wang, Xian Deng, Maolin Xu, Zimeng Zhu, Yuxin Liu, Herman W. Barkema, Eduardo R. Cobo, John P. Kastelic, Jian Gao, Bo Han

**Affiliations:** 1 College of Veterinary Medicine, China Agricultural University, Beijing, China; 2 College of Animal Science and Technology, Tarim University, Alar, Xinjiang, China; 3 Faculty of Veterinary Medicine, University of Calgary, Calgary, AB, Canada

**Keywords:** *Acinetobacter baumannii* ST3475, antimicrobial resistance, bovine mastitis, pathogenesis, whole genome sequencing

## Abstract

**Background:**

Bovine mastitis causes substantial economic losses worldwide. *Acinetobacter baumannii* has emerged as a pathogen in bovine mastitis, raising considerable concerns due to its multidrug resistance and potential zoonotic transmission. However, characteristics of *A. baumannii* involved in bovine mastitis in China remain poorly understood, particularly with emergence of novel sequence types.

**Methods:**

From 135 *Acinetobacter* spp. isolates isolated in China from bovine mastitis (2021-2024), we identified a novel sequence type, ST3475. Using the known bovine strain ST3256 for comparison, we conducted hybrid whole-genome sequencing, antimicrobial susceptibility testing, and multiple phenotypic pathogenicity assays (including biofilm formation, cell adhesion/invasion, and *in vivo* infection models).

**Results:**

The *A. baumannii* ST3475 chromosome is 3,875,774 bp with 1 plasmid (158,806 bp); it has an expanded repertoire of 70 virulence genes, including *csu* and *bap*, and 18 antimicrobial resistance genes. Furthermore, its genome contains a complement of phage elements, carbohydrate-active enzymes, and a unique carbapenemase gene *bla*_OXA-344_, indicating distinct evolution. Phenotypically, ST3475 demonstrated enhanced biofilm formation, adhesion, invasion, and cytotoxicity against bovine mammary epithelial cells. It also promoted secretion of IL-1β, IL-6, and TNF-α. In *Galleria mellonella*, the median lethal dose of ST3475 was 2 orders of magnitude lower than ST3256 (LD_50_ = 1,378,077 CFU/larva). In a murine mammary infection model, ST3475 caused substantial alveolar destruction and inflammatory infiltration.

**Conclusion:**

Phylogenetically, ST3475 belongs to clonal complex CC2 but represents a distinct, putative bovine-adapted lineage separate from major human clone CC1. Although currently susceptible to last-resort antimicrobials like carbapenems, carriage of high-risk resistance genes highlights livestock as a potential reservoir for clinically relevant resistance determinants. This study reveals ST3475 as a novel, virulent, and putative bovine-adapted lineage, underscoring the importance of One Health surveillance.

## Introduction

Bovine mastitis is the most common infectious disease of dairy cattle (Fadillah et al., 2025). Mammary gland infections caused by various pathogens decrease milk yield and quality and increase antimicrobial use and culling, resulting in substantial economic losses on dairy farms worldwide (Fadillah et al., 2025; Gonçalves et al., 2020). *Acinetobacter baumannii* is an important etiological factor in bovine mastitis, exacerbated by the diminishing efficacy of antibiotics with many antibiotic-resistant strains. In a comprehensive epidemiological survey across 15 large-scale dairy farms in 12 major milk-producing provinces of China, *Acinetobacter* spp. was isolated from 3.38% of 1,153 clinical mastitis milk samples, indicating a non-negligible prevalence with a recent trend toward increasing frequency (Song et al., 2020). In Egypt, *A. baumannii* strains were recently identified in 5.1% of subclinical mastitis milk samples (Abdel-Hameed et al., 2025). Although this genus encompasses several species, *A. baumannii* constitutes a clinically relevant proportion of these isolates, though comprehensive species-level surveillance in animal populations remains limited (van der Kolk et al., 2019). This emergence in cattle highlights a critical shift in the epidemiology of *A. baumannii* and raises important questions about its ecology, adaptation, and zoonotic potential.

The population structure and pathogenic mechanisms of *A. baumannii* in human infections have been extensively characterized. Based on multilocus sequence typing (MLST) schemes, a majority of clinically relevant, multidrug-resistant isolates belong to a limited number of globally disseminated clonal complexes (CCs), most notably CC1 and CC2 (Diancourt et al., 2010). In stark contrast, the genetic landscape of animal-associated *A. baumannii* appears distinct. Preliminary phylogenetic analyses suggest that strains isolated from cattle and other animals often cluster separately from the dominant human hospital lineages. In 1study, ~ 66.7% of bovine *A. baumannii* isolates were within CC2, but they often represented distinct and diverse STs not commonly isolated in human clinics, implying possible host-specific sub-lineages (Hamidian and Nigro, 2019; Boutzoukas and Doi, 2025). This phylogenetic separation suggests divergent evolutionary paths and adaptation to specific host niches. However, genomic determinants that facilitate colonization of the bovine mammary gland, mechanisms of pathogenicity in mastitis, and the real potential for cross-species transmission (either of the entire clone or, more likely, of mobile genetic elements carrying resistance or virulence genes) remain profoundly under-characterized. This constitutes an important knowledge gap within a One Health framework, which recognizes the interconnectedness of human, animal, and environmental health (Robinson et al., 2016; Maboni et al., 2020).

To bridge this gap, advanced genomic tools are indispensable. The advent of hybrid genome sequencing strategies, combining the high accuracy of short-read platforms (e.g., Illumina) with the long-range contiguity provided by long-read technologies (e.g., Oxford Nanopore or PacBio), has revolutionized bacterial genomics (Wick et al., 2017). This approach enables construction of complete, closed-genome assemblies, crucial for accurately identifying the genomic context of antimicrobial resistance genes (e.g., their location on plasmids, integrons, or transposons), delineating pathogenicity islands, and detecting structural variations that may be missed by short-read assemblies alone (De Maio et al., 2019; Chung et al., 2025). Furthermore, integrating such genomic data with hybrid sequencing and functional assays is key to transforming genetic associations into mechanistic insights. Following this model, studies of other pathogens have definitively linked specific gene systems (e.g., iron uptake to enhanced virulence traits like adhesion and biofilm formation), providing a validated strategy for investigating animal-adapted strains (Strzelecki and Nowicki, 2025; Azra et al., 2025; Borgio, 2025).

There are critical gaps in understanding genomic determinants, adaptive mechanisms, and pathogenic potential of novel, bovine-associated *A. baumannii* lineages, particularly within the Chinese context. To address these gaps, we isolated and characterized a novel *A. baumannii* sequence type, ST3475, from bovine mastitis. We hypothesized that ST3475 represents a distinct, putative bovine-adapted lineage with enhanced virulence and a unique resistance arsenal compared to both related bovine strains and dominant human clones. To test this hypothesis, we employed a hybrid sequencing strategy to obtain its complete genome and conducted a multifaceted comparison with a previously sequenced bovine mastitis *A. baumannii* isolate (ST3256) and representative human clinical strains. Specifically, this study aimed to: (1) delineate the unique genomic architecture of ST3475, including its resistome and virulome; (2) identify putative genetic markers of bovine host adaptation through comparative genomics; and (3) functionally validate its enhanced pathogenic potential using both *in vitro* and *in vivo* infection models. Our findings aim to elucidate adaptive strategies of *A. baumannii* in agricultural hosts and assess potential public health risks from a One Health perspective.

## Materials and methods

### Dairy herds, bacterial isolates and study design

This study utilized a national collection of 6,330 bovine mastitis milk samples from 21 provinces across China from 2021 to 2024. As part of this broader collection, 28 clinical mastitis milk samples were aseptically collected, following National Mastitis Council guidelines (Oliver et al., 2024), from individual quarters of affected cows in 2 focal herds between August 10 and 16, 2023. All milk samples were placed in 50 mL sterile tubes, transported on ice to the Mastitis Diagnostic Laboratory of China Agricultural University (Beijing), and processed for bacteriological culture. From the entire national collection, 135 *Acinetobacter* spp. isolates were recovered and stored. Furthermore, from the 28 samples collected in August 2023, 2 isolates of *A. baumannii* (ST3475 and ST3256, respectively) were identified and selected for detailed molecular and phenotypic characterization. Cows enrolled in the 2023 sub-study were characterized by parity (range 3-4), days in milk (range 150-200), and clinical severity (18 mild, 10 moderate). The herds had an average daily milk yield of 33.8 kg and 36.5 kg per cow and an average milk somatic cell count of 360,000 and 475,000 cells/mL, respectively. Furthermore, the cows were fed a total mixed ration, housed in freestall facilities with recycled manure solids used as bedding, and milked three times daily in a fishbone parlor. Pre- and post-milking teat disinfection was performed using 0.25% and 0.50% iodine-based solutions, respectively. Intramammary ceftiofur was routinely used for treatment of clinical mastitis. Bacterial growth was observed in 26 of the 28 milk samples, another had no growth, and the remaining 1 was considered contaminated due to the presence of >2 bacterial species.

### Bacterial culture conditions and bacterial analysis

All 135 *Acinetobacter* spp. isolates from the national collection were subjected to species identification and antimicrobial susceptibility testing. Bacteria were cultured on 5% sheep blood agar (BKMAM, Changde, Hunan, China) at 37 °C for 18–24 h. Initial identification was based on colony morphology and biochemical tests (indole, MR-VP, lactose fermentation, catalase, and motility), with final confirmation by 16S rRNA gene sequencing. For scanning electron microscopy (SEM, HITACHI, SU8100, Tokyo, Japan), bacterial cells were fixed with 1.25% glutaraldehyde, coated with gold, and imaged. MLST analysis was performed using the Oxford scheme with 7 housekeeping genes: cpn60, gdhB, gltA, gpi, gyrB, recA, and rpoD. The allelic profile of ST3475 was submitted and officially assigned by the PubMLST database (https://pubmlst.org/abaumannii/). MLST analysis revealed distinct STs. The 2 A*. baumannii* isolates (ST3475 and ST3256) recovered from the 2023 sub-study were further characterized by whole-genome sequencing and virulence factor profiling.

### Whole genome sequencing and library construction

For each isolate, genomic DNA was extracted using the TIANamp Bacteria DNA kit (Tiangen Biotech, Beijing, China) according to the manufacturer’s instructions. Quality, purity, integrality, and yield of the extracted genomic DNA were detected using 0.35% agarose gel electrophoresis and a NanoDrop2000 spectrophotometer (Thermo Fisher Scientific, Pittsburg, PA, USA), and quantified using a Qubit 4.0 fluorometer (Invitrogen, Carlsbad, CA, USA). Whole genome sequencing was performed on Illumina NovaSeq platform and the Oxford Nanopore Technologies (ONT) platform. For Illumina TruSeq Nano DNA LT sequencing, libraries were constructed by Shanghai Personal Biotechnology Co., Ltd. (Shanghai, China). After genome assembly, the whole-genome sequence of ST3475/ST3256 was compared to that of *A. baumannii* published using the NCBI Genbank nucleotide database (https://www.ncbi.nlm.nih.gov/). ST3475 was identified as a novel sequence type not deposited in the PubMLST database, whereas ST3256 has been previously characterized.

### Genome functional component analysis

Genome functional component analysis of ST3475/ST3256 was conducted utilizing GeneMarkS v4.32 (Besemer et al., 2001). Specifically, tRNAscan-SE (Chan et al., 2021), Barrnap (version 0.9), and Rfam (Kalvari et al., 2018) were employed for identification of tRNA, rRNA, and other non-coding RNAs (ncRNAs), respectively. Repeat sequences were analyzed via RepeatModeler software (Tempel, 2012), while the RepBase database was utilized to predict sequences homologous to known repeat sequences. Tandem repeat prediction was performed using TRF software (version 4.10.0). Additionally, CGview (Stothard and Wishart, 2005) was applied to provide a comprehensive overview of genome characteristics.

### Genome subsystem analysis

Genome subsystem analysis was conducted on ST3475/ST3256 to characterize relevant functional elements. Specifically, the Carbohydrate-Active Enzymes Database (CAZyme) (Lombard et al., 2014) was employed for prediction of carbohydrate-active enzymes, while the Virulence Factor Database (VFDB) (Liu et al., 2019) and the Comprehensive Antibiotic Resistance Database (CARD) (Alcock et al., 2020) were utilized to retrieve virulence-related and pathogenicity genes, respectively.

### Genome functional annotation analysis

Multiple specialized databases and tools were applied to analyze functions of protein-coding genes in ST3475/ST3256. These included the Non-Redundant Protein Database (NR) (Wu et al., 2002), the Cluster of Orthologous Groups of proteins (COG) (Powell et al., 2014), and PhiSpy software (Akhter et al., 2012) - the latter was used specifically for prophage prediction. Additional databases involved in the annotation process were the Transporter Classification Database (TCDB), the Pathogen-Host Interactions Database (PHI), the Kyoto Encyclopedia of Genes and Genomes (KEGG) (Moriya et al., 2007), and Gene Ontology (GO) (Conesa and Gotz, 2008).

### Antimicrobial resistant testing

Antimicrobial susceptibility profiles of ST3475 and ST3256 were assessed by the Kirby-Bauer disk diffusion method according to Clinical and Laboratory Standards Institute guidelines (CLSI M100, 35^th^ Edition) using Mueller-Hinton agar (Haibo, Qingdao, Shandong, China). A total of 10 antimicrobial agents belonging to 6 classes were tested, included piperacillin (100 μg), ceftazidime (30 μg), ceftriaxone (30 μg), imipenem (10 μg), amikacin (30 μg), gentamicin (10 μg), minocycline (30 μg), ciprofloxacin (5 μg), levofloxacin (5 μg), and cotrimoxazole (25 μg) (BKMAM, Changde, Hunan, China). After incubation at 37 °C for 18–24 h, inhibition zone diameters were measured and the results were interpreted as susceptible (S), intermediate (I), or resistant (R) based on CLSI breakpoints. All tests were performed in triplicate.

### Biofilm formation assay

Biofilm formation was assessed using the crystal violet staining method. Bacteria were inoculated into 96-well microtiter plates containing Luria-Bertani (LB) broth and incubated at 37 °C for 24 h. The supernatant was discarded, and wells were washed three times with phosphate buffer saline (PBS). After drying, 1% crystal violet solution was added to each well and incubated for 30 min. Excess crystal violet was washed away and plates were dried. The biofilm was solubilized with 95% ethanol and absorbance at 570 nm (OD_570_) was measured.

### Growth curve of *A. baumannii*

Growth curves of *A. baumannii* ST3475 and ST3256 were assessed simultaneously. For each isolate, 3 mL of bacterial fluid was diluted in 60 mL LB and well mixed. Thereafter, sterile tubes with 4-mL aliquots were placed on a constant temperature shaker (37 °C, 220 rpm). Every 2 h, from 0 to 36 h, 1 tube per isolate was removed and optical density (OD) of bacterial suspension was determined at 600 nm in a UV spectrophotometer.

### Bovine mammary epithelial cells infection model for *A. baumannii*

Lactate dehydrogenase (LDH) release assay: bMECs were used at passages 3-5, cultured in DMEM with 10% FBS. The bMECs were seeded in 96-well plates, infected with *A. baumannii* (MOI = 10, justified by physiological relevance and previous studies) for 24 h. Supernatants were collected, and LDH activity was detected using an LDH Assay Kit (Beyotime, Shanghai, China) following the manufacturer’s instructions, with absorbance measured at 450 nm by a microplate reader. Bacterial adhesion assay: Confluent bMECs monolayers in 24-well plates were infected with *A. baumannii* (MOI = 10) for 1 and 2 h at 37 °C with 5% CO_2_, washed thrice with PBS (0.01 M, pH 7.4; Servicebio, Wuhan, Hubei, China) to remove non-adherent bacteria, lysed with 0.1% Triton X-100, and lysates were serially diluted, plated on LB agar plates (Aoboxin, Beijing, China) and incubated at 37 °C for 24 h for colony-forming unit (CFU) counting. For invasion assays, bMECs were infected with *A. baumannii* (MOI = 10) for 1 and 2 h. Extracellular bacteria were then killed by 1 h incubation with 100 μg/mL gentamicin, a treatment validated to be fully lethal extracellularly but non-toxic intracellularly. Following cell lysis, intracellular bacteria were enumerated by CFU counting (Yousefi et al., 2024).

### Inflammatory cytokines ELISA assay

Supernatants of infected bMECs were collected at 0, 3, 6, 9, and 12 h post-infection; concentrations of inflammatory cytokines (tumor necrosis factor-α (TNF-α), interleukin-1β (IL-1β), interleukin-6 (IL-6)) were measured using specific ELISA Kits (MLE, Wuhan, Hubei, China) according to the manufacturer’s protocols, with absorbance recorded at 450 nm and cytokine concentrations calculated via standard curves.

### Galleria mellonella infection model for *A. baumannii*

Final-instar *G. mellonella* larvae (weight: 200–300 mg) were used to evaluate *A. baumannii* pathogenicity, including survival rate, median lethal dose (LD_50_) and melanization level. *A. baumannii* was cultured to logarithmic phase, harvested by centrifugation, and resuspended in sterile PBS. The bacterial suspension was serially diluted to prepare 6 gradient concentrations (3×10^4^, 1×10^5^, 3×10^5^, 1×10^6^, 3×10^6^, and 1×10^7^ CFU/10 μL). Larvae were randomly divided into 7 groups (10 larvae per group): 6 infection groups and 1 control group. The survival and mortality rates of larvae were recorded at 12, 24, 36, 48, 60, and 72 h post-infection.

### Murine mammary infection model for *A. baumannii*

Female BALB/c mice (6–8 wk, Merial-Vital Laboratory Animal Technology, Beijing, China) were used to determine the pathogenic role of *A. baumannii* during intramammary infection. Pregnant (20 d of gestation) mice were kept in germfree isolators and fed *ad libitum* in a controlled environment with light and dark cycles (12 h light and 12 h darkness). On the third day after parturition, mice were anesthetized with an intramuscular injection of 50 mg/kg Zoletil50 (Virbac, Carros, France). Ducts of the fourth pair of mammary glands were exposed by cutting the teat tip and 100 μL of bacterial suspension (10^6^ CFU) was slowly injected using a gauge blunted needle (30 G, 25 mm) (Hamilton Company, Reno, NV), n=6 mice per group. The pups were removed 2 h before intramammary inoculation. The sedated mice were euthanized by cervical dislocation and dissected. The skin was fixed with pins before photographing the mammary glands. Bacterial burden in mammary glands was quantified by CFU counting. Mammary gland tissue was fixed in 4% paraformaldehyde, embedded, sectioned, and stained with hematoxylin-eosin. Histological examination was conducted to evaluate necrosis, neutrophilic inflammation, and lymphocytic infiltration, using a predefined scoring system for inflammation, necrosis and percent of tissue affected (Caddey et al., 2025). Randomization and blinding were applied to all histological assessments to ensure objectivity.

### Statistical analysis

Data are presented as mean ± standard deviation (SD) from at least 3 independent experiments. Analyses were performed using SPSS 26.0 (IBM Corp., USA), with graphs generated using GraphPad Prism 8.0 (GraphPad Software, USA). Statistical analyses were performed using GraphPad Prism. For *in vitro* samples (n=3), non-parametric Kruskal-Wallis test followed by pairwise Wilcoxon rank-sum test with Bonferroni correction was used. For *G. mellonella* (n=10) and mice (n=6), one-way ANOVA followed by Tukey’s multiple comparisons test was applied. Survival curves from *G. mellonella* experiments were analyzed using the Kaplan-Meier method and compared with the log-rank test. For antimicrobial susceptibility, categorical interpretations (susceptible, intermediate, resistant) were based on CLSI breakpoints. Significance levels were set at *P < 0.05, **P < 0.01, and ***P < 0.001.

## Results

### Identification and MLST typing of *A. baumannii* ST3475

**Figure 1:**
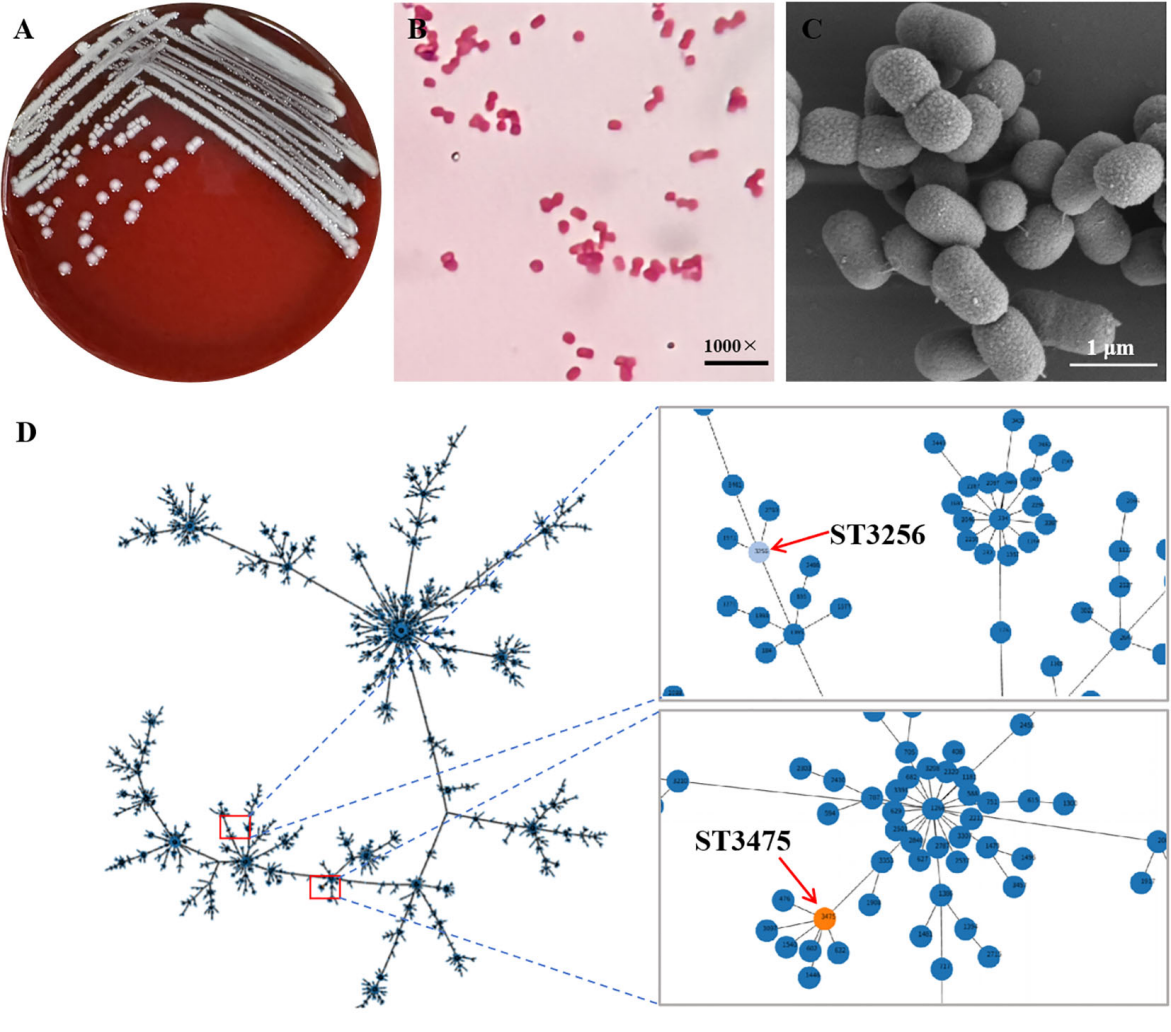
Combined identification and genotyping of *A. baumannii.***(A)** Complex streak of *A. baumannii* colonies grown on sheep blood agar (24 h, 37 °C) with white, protuberant, smooth measuring 1.5–2.0 mm in diameter; **(B)** Gram staining of *A. baumannii*: Gram-negative short rods, 1000× magnification; **(C)** SEM of *A. baumannii* (scale bar = 1 μm) cells coated rough surfaces with pili structures and extracellular polymeric substances; and **(D)** Minimum spanning tree of *A. baumannii* isolates, showing clonal relationships of sequence types (ST3256 and ST3475) and belonging to clonal complex 2 (CC2).

From 2021 to 2024, a total of 135 *Acinetobacter* spp. isolates (2.1%) were recovered from 6,330 bovine mastitis milk samples across 21 provinces in China. *A. baumannii* accounted for 10.4% (n=14) of these. From the dedicated sampling of 28 clinical cases in August 2023, 2 isolates of *A. baumannii* were cultured, representing an incidence of 7.1% (2/28) within that cohort. These 2 isolates, identified as ST3475 and ST3256, were selected for further genomic analysis. *A. baumannii* formed white, circular, moist, smooth colonies measuring 1.5–2.0 mm in diameter on blood agar plates (**Figure 1A**), Gram staining revealed red coccobacillus under 1000-X magnification, confirming it as a Gram-negative coccobacillus (1.2–1.8 × 0.8–1.0 μm) (**Figure 1B**). SEM showed cells with rough surfaces, pili structures, extracellular polymeric substances, and short rods (0.8-1.0 × 1.2-1.8 μm) (**Figure 1C**). Biochemical tests were indole-positive, catalase-positive, lactose-negative, non-motile (data not shown). MLST analysis revealed distinct STs. The allele profiles for ST3475 were ^Oxf^cpn60 = 1, ^Oxf^gdhB=42, ^Oxf^gltA=35, ^Oxf^gpi=103, ^Oxf^gyrB=50, ^Oxf^recA=49, and ^Oxf^rpoD=5, whereas for ST3256 it was ^Oxf^cpn60 = 4, ^Oxf^gdhB=59, ^Oxf^gltA=36, ^Oxf^gpi=279, ^Oxf^gyrB=34, ^Oxf^recA=28, and ^Oxf^rpoD=4 (**Table 1**). Phylogenetic clustering in the minimum spanning tree demonstrated that ST3475 and ST3256 occupy distinct clades within clonal complex 2 (CC2) (**Figure 1D**). This complex is genetically distinct from the predominant human clinical lineage CC1 (typically ST2), differing by at least 5 alleles. The unique allele combination defining ST3475 confirms its status as a novel sequence type and suggests it represents a divergent, putative bovine-adapted sub-lineage. ST3475 was officially assigned by PubMLST under the Oxford scheme.

**Table 1:** Multilocus sequence typing allele profiles o*f* 2 isolates of *A. baumannii*.

Item	ST 3475	ST3256
Province	Inner Mongolia	Inner Mongolia
^Oxf^cnp60	1	4
^Oxf^gdhB	42	59
^Oxf^gltA	35	36
^Oxf^gpi	103	279
^Oxf^gyrB	50	34
^Oxf^recA	49	28
^Oxf^rpoD	5	4
MLST	3475	3256

### Chromosome and plasmid characteristics of *A. baumannii* ST3475

**Figure 2:**
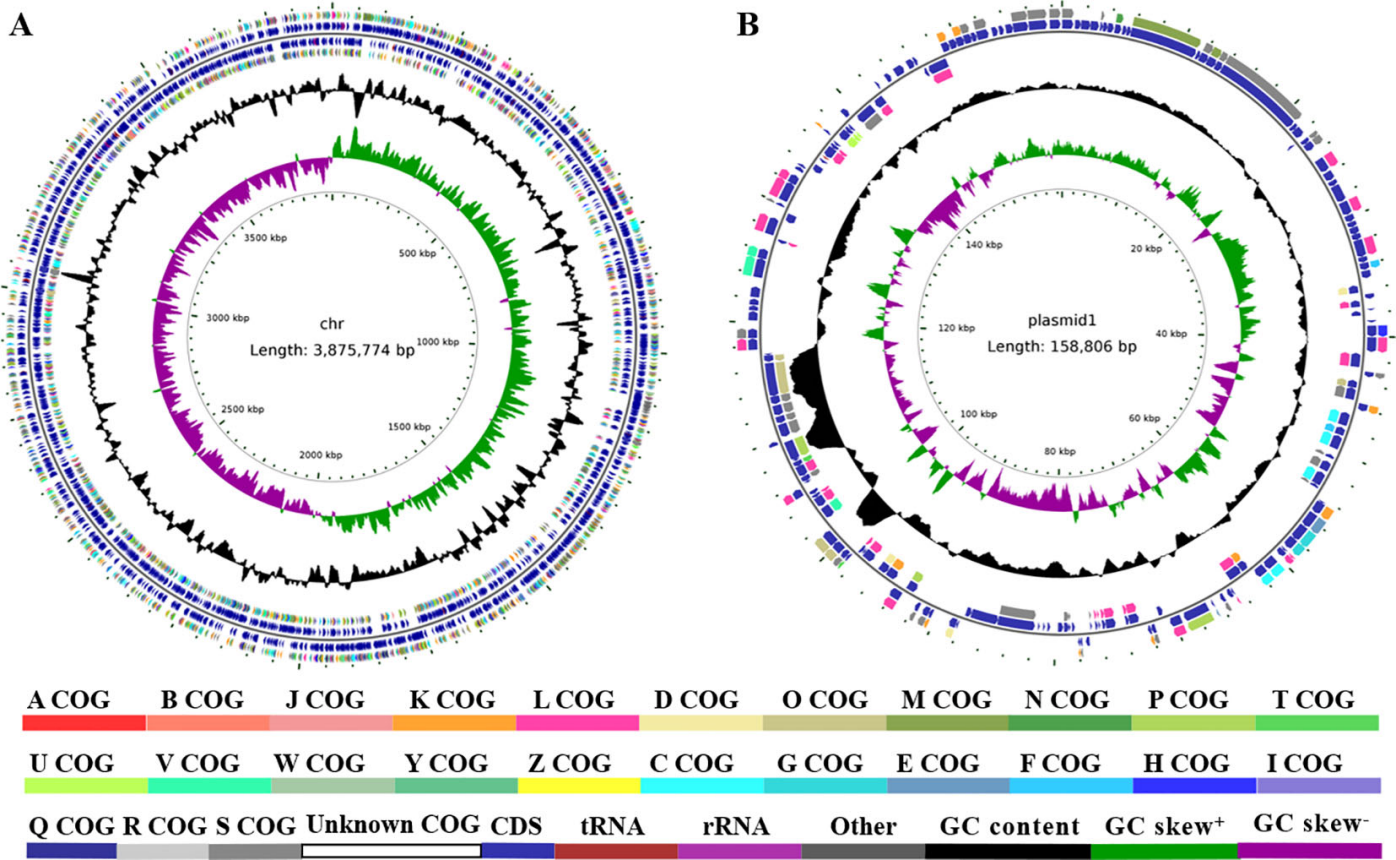
Genomic architecture of *A. baumannii* ST3475. **(A)** ST3475 Chromosome circular genome plot showing (from outer to inner): forward strand CDSs, reverse strand CDSs (colored by COG categories), G+C content (black line), G+C skew (purple/green), annotated resistance genes (red), virulence genes (blue), iron acquisition systems (orange), and repeat sequences (gray); and **(B)** ST3475 Plasmid predicted genomic islands with high-confidence regions (score ≥ 0.80) highlighted in red.

The chromosome of ST3475 was 3,875,774 bp in length with a GC content of 39.04%, encoding 3,586 ORFs, 74 tRNAs, 18 rRNAs, and 39 ncRNAs. In contrast, the chromosome of ST3256 was shorter (3,733,226 bp) with a slightly higher GC content (39.09%), containing 3,477 ORFs, 74 tRNAs, 18 rRNAs, and 35 ncRNAs (**Table 2**, **Figure 2A**). Regarding plasmids, ST3475 harbored a single plasmid of 158,806 bp with a GC content of 40.81%, encoding 176 ORFs, 1 tRNA, 1 ncRNA, and 1 prophage (**Table 2**, **Figure 2B**).

**Table 2:** General features of 2 isolates of *A. baumannii*, based on whole-genome sequencing.

Genomic parameter	ST3475	ST3256
Chromosome		
Accession no.	JBSLMF000000000	JBSLMG000000
Sequence length/bp	3 875 774	3 733 226
GC content/%	39.04	39.09
ORF no.	3 586	3 477
tRNA copy no.	74	74
rRNA copy no.	18	18
ncRNA copy no.	39	35
Prophage no.	25	22
Short interspersed repeats no.	7	13
Long interspersed repeats no.	21	31
Long terminal repeats no.	63	83
Transposons no.	45	43
Unclassified interspersed Repeats no.	10	11
Satellites RNA no.	5	3
Simple-repeats no.	0	0
Plasmid		
Sequence length/bp	158 806	Plasmid 1-7: 142 221, 12 024, 7 061, 5 078, 3 503, 2 924, 2 278
GC content/%	40.81	Plasmid 1-7: 40.76, 35.87, 32.32, 39.37, 37.94, 36.18, 39.86
ORF no.	176	Plasmid1-7: 141, 11, 10, 4, 2, 3, 2
tRNA copy no.	1	1(Plasmid 1)
rRNA copy no.	0	0
ncRNA copy no.	1	1(Plasmid 1)
Prophage no.	1	2(Plasmid 1)
Short interspersed repeats no.	0	0
Long interspersed repeats no.	0	0
Long terminal repeats no.	0	0
Transposons no.	0	0
Unclassified interspersed Repeats no.	0	0
Satellites RNA no.	0	0
Simple-repeats no.	0	0

ORF, open reading frame; ncRNA, non-coding RNA.

^a^ Based on NCBI prokaryotic genomic annotation pipeline.

### Genome subsystem analysis

**Figure 3:**
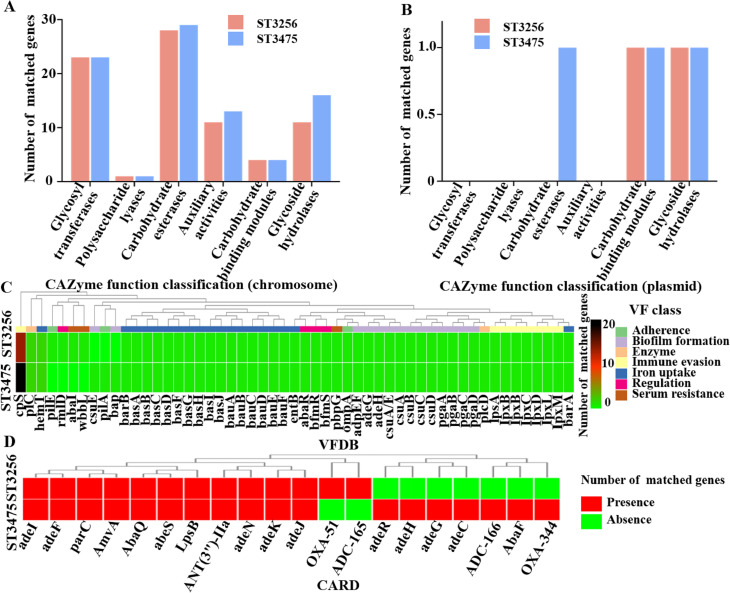
CAZyme, virulence factor, and antimicrobial resistance gene profiles of *A. baumannii*. **(A)** Virulence factor annotations (from VFDB) of *A. baumannii* ST3256 and ST3475, categorized by virulence function; **(B)** CAZyme functional classification of *A. baumannii* based on chromosomal genes; **(C)** CAZyme functional classification of *A. baumannii* based on plasmid-borne genes; and **(D)** Antimicrobial resistance gene profiles (from CARD) of *A. baumannii* ST3256 and ST3475, showing presence/absence of resistance determinants.

Prophages of *A. baumannii* were predicted using the PHASTER tool. ST3475 contained more prophages (25 on the chromosome and 1 on the plasmid) compared to ST3256 (22 on the chromosome and 2 on the plasmids) (**Table 2**). CAZyme analysis revealed 86 enzymes in the ST3475 chromosome, including 23 glycosyltransferases (GTs), 1 polysaccharide lyase (PL), 29 carbohydrate esterases (CEs), 13 auxiliary activities (AAs), 4 carbohydrate binding modules (CBMs) and 16 glycoside hydrolases (GHs), plus 3 plasmid-borne CAZymes. Furthermore, the ST3256 chromosome contained 23 GTs, 1 PL, 28 CEs, 11 AAs, 4 CBMs and 11 GHs, with 2 plasmid-borne CAZymes (**Figures 3A, B**). VFDB analysis revealed a total of 49 types comprising 70 virulence factors (VFs) in ST3475, and 50 types comprising 65 VFs in ST3256, respectively. The VFs were classified into adherence, biofilm formation, enzyme, immune evasion, iron uptake, regulation, and serum resistance. ST3475 had more VFs associated with biofilm formation, iron uptake, and serum resistance compared to ST3256 (**Figure 3C**). Antibiotic resistance gene screening via the CARD identified 18 resistance genes in ST3475 versus 13 in ST3256 (**Figure 3D**), encompassing determinants for β-lactamase genes (*blaOXA-51*, *blaOXA-344, blaADC-166*), efflux pumps (*AbaF, adeN*, *AbaQ*) and aminoglycoside (*ANT(3”)-Ila, AmvA, adeR, adeF*, *adeG, adeH, adeI*), tetracyclines (*adeJ, adeK*), macrolides (*abeS*), Polymyxins (*LpsB*), Fluoroquinolones (*parC*).

### Genome functional annotation analysis

**Figure 4:**
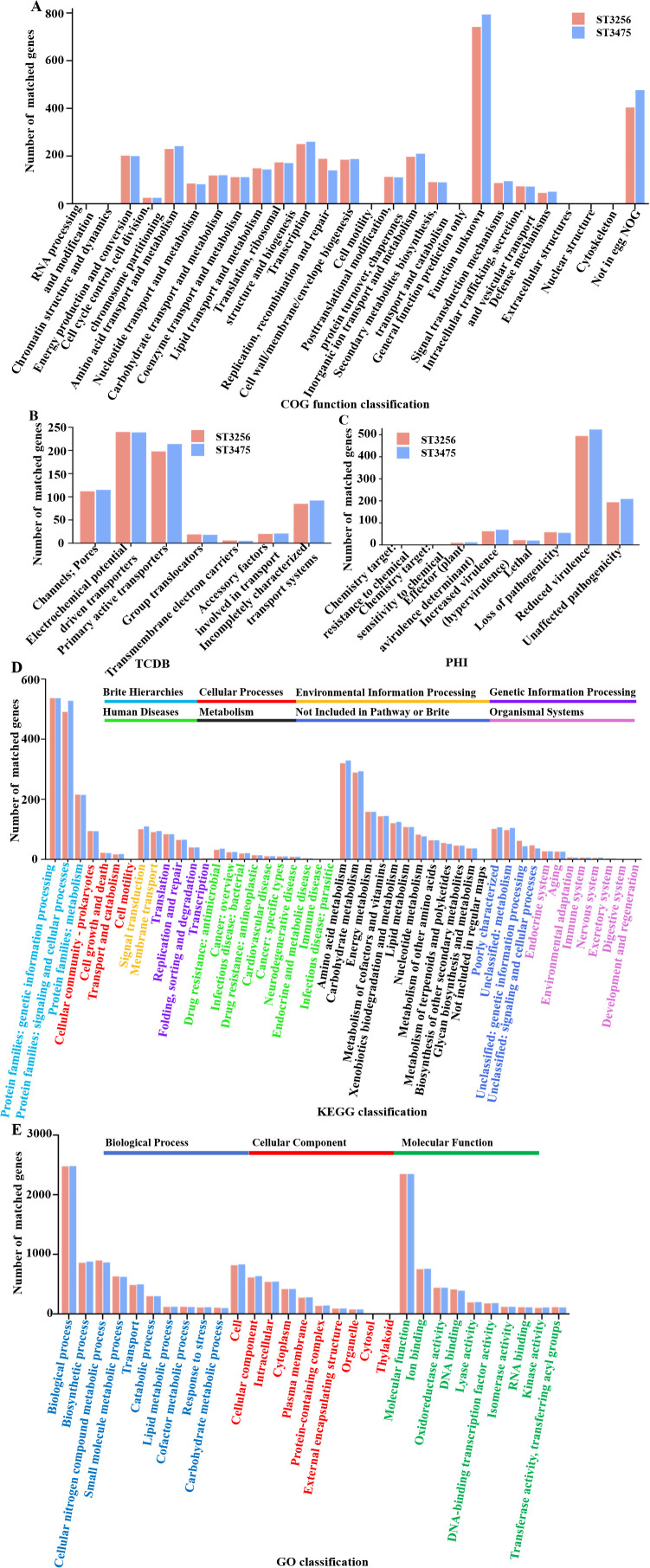
Functional annotation and classification of genomic features in *A. baumannii.***(A)** COG functional classification of *A. baumannii* genes; **(B)** TCDB classification of *A. baumannii* transporter-related genes; **(C)** PHI database annotation of *A. baumannii* genes; **(D)** Functional annotation of *A. baumannii* genes, including protein families, KEGG pathway classification; and **(E)** Detailed GO classification of *A. baumannii* genes, categorized into biological process, cellular component, and molecular function.

Functional annotation of the ST3475 genome assigned 3,578 chromosomal genes in the NR database, compared to 3,472 for ST3256 (**Table 3**). COG analysis confirmed enrichment in membrane transport, signal transduction, and carbohydrate metabolism with 3109 genes (**Figure 4A**). TCDB identified 704 transporter genes in ST3475, with a notable increase in primary active transporters and incompletely characterized transport systems compared to 680 genes to ST3256 (**Figure 4B**). KEGG pathway annotation 1907 highlighted enrichment genes in metabolic pathways (**Figure 4D**), whereas PHI-base analysis identified 754 genes in ST3475 associated with pathogen-host interactions, indicating enhanced interactive capacity, compared to 725 genes in ST3256 (**Figure 4C**). GO terms revealed higher gene counts in biological processes and molecular functions for ST3475 (**Figure 4E**).

**Table 3:** Functional annotation (number of protein-coding genes) in chromosome of 2 isolates of *A. baumannii*.

Annotation in database	ST3475	ST3256
NR	3 578	3 472
COG	3 109	3 073
TCDB	704	680
KEGG	1 917	1 907
PHI	754	725
GO	2 619	2 619

### Antimicrobial resistance profiles of *A. baumannii* isolate from bovine mastitis samples

Antimicrobial resistance profiles of *A. baumannii* ST3475 isolate against 10 agents revealed that both ST3475 and ST3256 are multidrug-resistant, defined as resistance to ≥ 3 antimicrobial classes beyond intrinsic resistance. Both strains were intrinsically resistant to piperacillin, ceftazidime and ceftriaxone. ST3475 had broader acquired resistance, including to aminoglycosides and gentamicin. ST3475 also had intermediate susceptibility to ciprofloxacin, whereas ST3256 was susceptible (**Table 4**).

**Table 4:** Susceptibility of 2 isolates of *A. baumannii* to antimicrobial drugs.

Antimicrobial class	Compound	ST3475		ST3256		Interpretive criteria
		Inhibition zone diameter/mm	Susceptibility	Inhibition zone diameter/mm	Susceptibility	*Acinetobacter* spp.
β-lactams	Piperacillin	16.00 ± 1.00	R	16.67 ± 0.58	R	S: ≥21;I: 18-20;R: ≤17
	Ceftazidime	13.83 ± 0.29	R	13.83 ± 0.76	R	S: ≥17;I: 15-16;R: ≤14
	Ceftriaxone	8.83 ± 0.29	R	2.83 ± 0.29	R	S: ≥21;I: 14-20;R: ≤13
Carbapenems	Imipenem	23.83 ± 0.29	S	22.67 ± 0.58	S	S: ≥22;I: 19-21;R: ≤18
Aminoglycosides	Amikacin	19.50 ± 0.50	S	16.17 ± 0.29	I	S: ≥17;I: 15-16;R: ≤14
	Gentamicin	11.33 ± 0.29	R	13.67 ± 0.58	I	S: ≥15;I: 13-14;R: ≤12
Tetracyclines	Minocycline	20.33 ± 0.29	I	20.33 ± 0.58	I	S: ≥22;I: 18-21;R: ≤17
Fluoroquinolones	Ciprofloxacin	20.50 ± 0.50	I	22.33 ± 0.58	S	S: ≥21;I: 16-20;R: ≤15
	Levofloxacin	20.50 ± 0.50	S	22.83 ± 0.29	S	S: ≥17;I: 14-16;R: ≤13
Folate pathway inhibitors	Cotrimoxazole	16.00 ± 1.00	S	16.17 ± 1.04	S	S: ≥16;I: 11-15;R: ≤10

*S*: susceptible, *I*: intermediate susceptible, *R*: resistant.

### Enhanced pathogenic properties *in vitro* and *in vivo*

**Figure 5:**
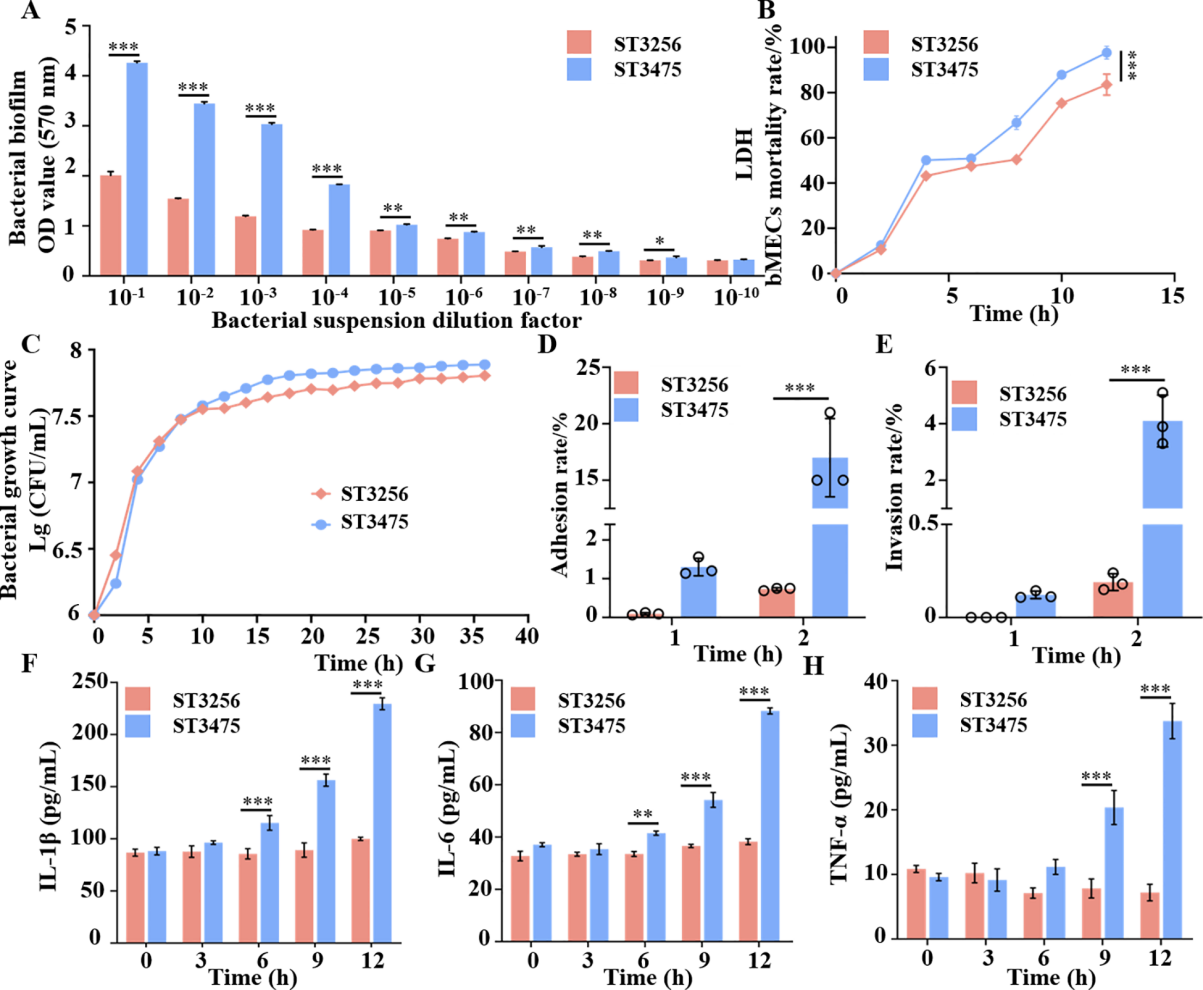
Phenotypic and pathogenicity analyses of *A. baumannii*: Biofilm formation, cytotoxicity, growth, and inflammatory responses. **(A)** Biofilm formation of *A. baumannii* at various dilutions; **(B)** LDH release of *A. baumannii* over time; **(C)** Growth curves of *A. baumannii* ST3256 and ST3475; **(D)** Adhesion rate of *A. baumannii* to host cells at various time points; **(E)** Invasion rate of *A. baumannii* into host cells at various time points; **(F)** Secretion of IL-1β induced by *A. baumannii* over time; **(G)** Secretion of IL-6 induced by *A. baumannii* over time; and **(H)** Secretion of TNF-α induced by *A. baumannii* over time. Significance levels: *P < 0.05; **P < 0.01; ***P < 0.001.

**Figure 6:**
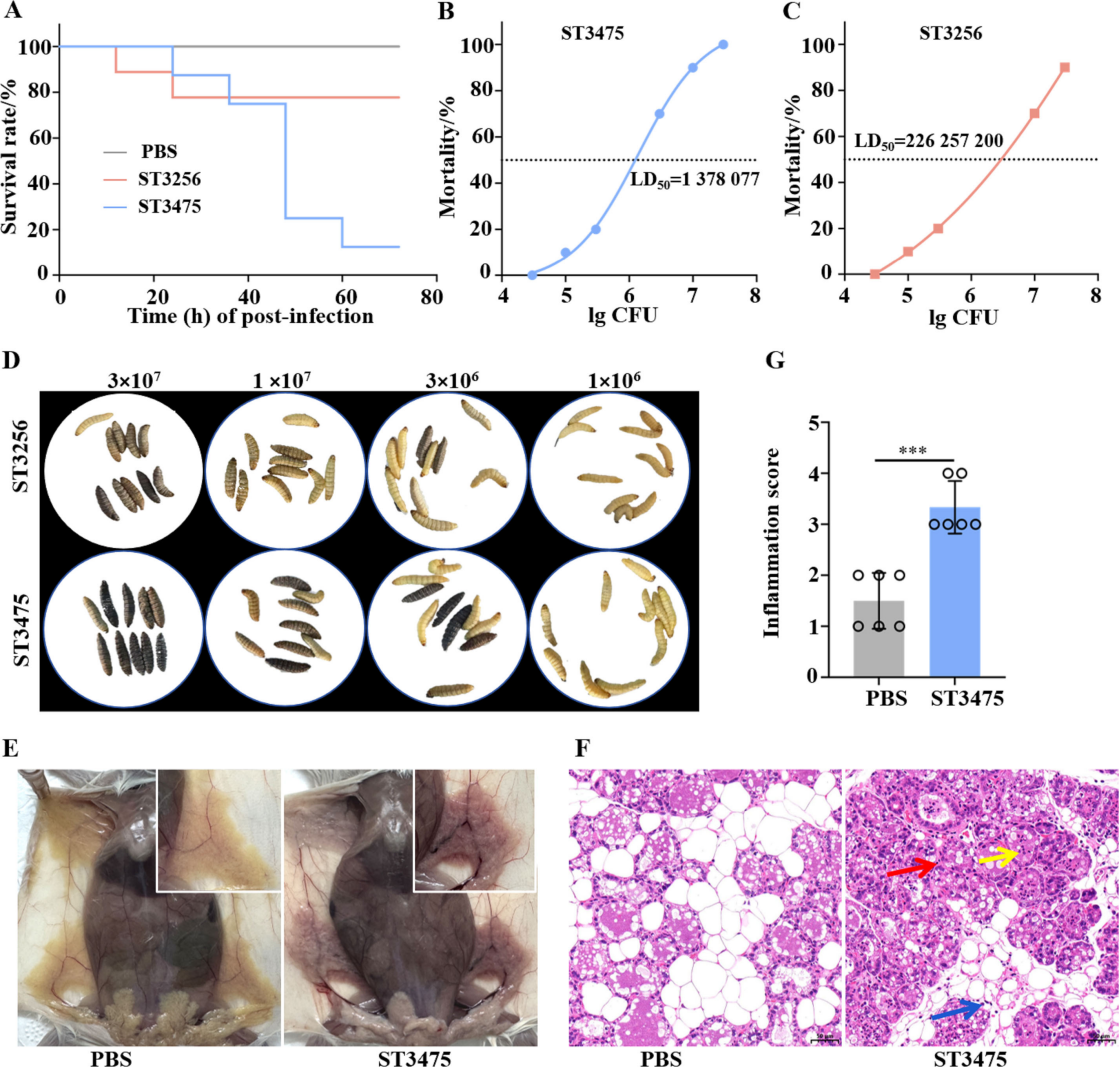
Pathogenicity evaluation of *A. baumannii* in *Galleria mellonella* and in murine mammary infection models. **(A)** Survival curve of *G. mellonella* larvae post-infection with *A. baumannii* ST3256, ST3475; **(B)** LD_50_ determination of *A. baumannii* ST3475 in *G. mellonella*; **(C)** LD_50_ determination of *A. baumannii* ST3256 in *G. mellonella*; **(D)** Melanization response of *G. mellonella* larvae infected with *A. baumannii* ST3256/ST3475 at various bacterial doses; **(E)** Gross appearance of mammary tissue damage in mice infected with *A. baumannii* ST3475; **(F)** H&E stained murine mammary tissue post-infection with *A. baumannii* ST3475; and **(G)** Inflammation score of murine mammary tissue post-infection with *A. baumannii* ST3475. Significance levels: ***P < 0.001.

*In vitro* assays demonstrated that ST3475 had enhanced pathogenic traits relative to ST3256. It produced 2.1-fold more biofilm at optimal dilution (*P* < 0.001; **Figure 5A**), consistent with its intact *csu* operon. ST3475 also induced higher lactate dehydrogenase release from host cells (*P* < 0.01; **Figure 5B**), exhibited faster proliferation with a shorter lag phase and higher maximum optical density (**Figure 5C**), and had approximately two-fold greater adhesion (*P* < 0.05; **Figure 5D**) and three- to four-fold higher invasion efficiency into bovine mammary epithelial cells (*P* < 0.01; **Figure 5E**). Furthermore, ST3475 triggered a stronger inflammatory response, inducing significantly higher concentrations of IL-1β, IL-6, and TNF-α at 9 h post-infection (**Figures 5F–H**). Furthermore, *in vivo Galleria mellonella* infection models confirmed that ST3475 exhibited a lower LD_50_ than ST3256 (**Figures 6A–C**), causing 90% mortality within 60 h at 10^7^ CFU/larva compared to 20% for ST3256. ST3475 also suppressed larval melanization by 56.7% (**Figure 6D**), indicating superior immune evasion. In a murine mammary infection model, ST3475 (1×10^6^ CFU/gland) induced severe mastitis within 24 h, characterized by marked gland enlargement, vascular congestion, and hemorrhage (**Figure 6E**). The mean bacterial load in mice of the ST3475 group was 2.4×10^6^ CFU/g after infection, compared to 0 CFU/g in the PBS control group. Histopathological examination revealed extensive neutrophilic infiltration, alveolar destruction, edema, and epithelial necrosis (**Figure 6F**), with higher inflammation scores compared to PBS (*P* < 0.001; **Figure 6G**).

## Discussion

*A. baumannii* has been increasingly recognized as an important pathogen in bovine mastitis across diverse geographical regions. An early study from South Korea reported *A. baumannii* in 7.6% of Gram-negative bovine mastitis isolates (Nam et al., 2010). Subsequently, studies from India (Ranjani et al., 2022), Brazil (de Oliveira et al., 2022), and most recently Egypt (Abdel-Hameed et al., 2025) have identified this pathogen in 5.1–5.5% of subclinical and environmental mastitis cases. In line with this global trend, in our investigation, *A. baumannii* accounted for 10.4% of *Acinetobacter* spp. recovered from bovine mastitis in China, indicating a notable and concerning prevalence. Compounding this issue, *A. baumannii* isolated from such environments frequently exhibits resistance to nearly all first-line antibiotics, highlighting a substantial challenge for mastitis management and antimicrobial stewardship.

### Genomic uniqueness and adaptive profile of *A. baumannii* ST3475

This study provides the first comprehensive characterization of *A. baumannii* ST3475, a novel and virulence lineage isolated from bovine mastitis. The pathogen exhibited a distinctive genomic profile suggestive of putative adaptation to the bovine mammary niche; this was based on association and phylogenetic clustering rather than formal pangenome or gene enrichment analysis while carrying elements of public health concern. Phylogenetically, ST3475 belongs to CC2 but is distinguished from major human-associated clones within this complex, such as ST2, by a unique allele profile (Holt et al., 2019; Słoczyńska et al., 2021). This genetic divergence suggests a separate evolutionary trajectory, potentially driven by adaptation to the livestock environment. Genomically, ST3475 has an expanded arsenal of 70 virulence genes, with notable enrichment in iron acquisition systems critical for survival in the iron-limited mammary gland (Artuso et al., 2023). Its genome also exhibits significant plasticity, harboring 25 prophages regions and numerous mobile genetic elements, which together constitute 6.06% of its chromosome, indicating a history of extensive horizontal gene transfer (Tiku, 2022). This genomic architecture likely facilitates rapid adaptation.

### Contrasting *A. baumannii* ST3475 with dominant human clinical lineages

Key differences distinguish ST3475 from globally disseminated human clinical lineages, particularly CC1. Whereas human CC1 strains are optimized for hospital settings and often exhibit pan-drug resistance (Wong et al., 2017; Hamidian and Nigro, 2019; Zhang et al., 2022), ST3475’s genomic fine-tuning appears directed toward bovine host colonization. A critical distinction lies in its antimicrobial resistance profile. Phenotypically, ST3475 remains susceptible to last-resort agents like carbapenems (imipenem) and fluoroquinolones, to which human multidrug-resistant isolates are commonly resistant (Magiorakos et al., 2012). However, a major point of convergence and concern is carriage of the high-risk carbapenemase gene *bla*OXA-344 on the chromosome; perhaps the absence of phenotypic carbapenem resistance is explained by low expression or the requirement for specific activation conditions. Although not phenotypically expressed as carbapenem resistance in this isolate, its presence in a livestock reservoir is alarming, as mobile genetic elements can facilitate its transfer to human pathogens, bridging ecological spheres (Noel et al., 2022). This juxtaposition-current phenotypic susceptibility versus the carriage of potent resistance determinants highlights livestock as a potential reservoir for the emergence of resistance.

### Enhanced virulence and resistance relative to the bovine isolate *A. baumannii* ST3256

Given that only 2 bovine isolates were compared, conclusions of enhanced virulence and resistance are limited to these 2 strains and may not represent all bovine-associated *A. baumannii* lineages. Compared to ST3256, ST3475 demonstrates a consistently more formidable profile. Genomically, ST3475 harbors a broader complement of antimicrobial resistance genes (18 vs. 13 in ST3256) and virulence factors (70 vs. 65). Critically, ST3475 possesses an intact *csuA/BABCDE* pilus operon and the biofilm-associated protein gene *bap*, which are absent or incomplete in ST3256. These genetic differences may directly underpin the enhanced pathogenic phenotypes observed. The intact *csu* system is strongly linked to the approximately two-fold greater adherence of ST3475 to bMECs, a critical first step in infection (Álvarez-Fraga et al., 2016; Ahmad et al., 2023). Similarly, the presence of *bap* correlates with the 1.9-fold increase in biofilm formation, enhancing environmental persistence and antibiotic tolerance (Zeighami et al., 2019). Phenotypic resistance also diverged; ST3475 displayed resistance to streptomycin and cefuroxime axetil, whereas ST3256 was intermediate or susceptible, correlating with the presence of corresponding resistance genes like *aph(3’)-VI* and an expanded β-lactamase repertoire.

### One health implications, limitations, and future directions

The discovery of ST3475 has important One Health implications. Identification of *bla*OXA-344 on the chromosome within a bovine isolate underscores livestock as a potential reservoir for globally prevalent carbapenemases (Bhargavi et al., 2025). Notably, although *bla*OXA-344 is classified as a carbapenemase gene, the isolate remains phenotypically susceptible to imipenem. We hypothesize that this discrepancy may result from the absence of a strong promoter or positive regulatory elements driving *bla*OXA-344 expression. In support of this regulatory model, previous studies demonstrated that expression of virulence and resistance genes can be silenced by the lack of critical transcriptional regulators, such as the iron-sulfur (Fe-S) cluster regulator IscR, which directly controls expression of multiple virulence and metabolic loci in pathogenic bacteria (Balderas et al., 2021). Thus, *bla*OXA-344 in ST3475 may be maintained in a low-expression or transcriptionally silent state without sufficient activation signals or regulatory factors, leading to the lack of phenotypic carbapenem resistance. Quantitative expression analysis and promoter characterization in future work will further clarify this mechanism. Mobile genetic elements can facilitate the transfer of such high-risk determinants to human-adapted clones, emphasizing the necessity of integrated, cross-sectoral surveillance to monitor gene flow (Scoffone et al., 2025; Noel et al., 2022).

Key limitations of this study were the single geographic origin, comparison with only 1 bovine isolate (ST3256), lack of gene knockout and complementation for virulence determinants, and using a murine model that cannot fully replicate bovine mastitis. Therefore, future studies should include pangenome analysis, gene functional validation, and bovine primary cell models. Furthermore, although the murine mammary infection model confirmed severe pathogenic potential, due to anatomical and immunological differences between murine and bovine mammary glands, the model cannot fully recapitulate all aspects of chronic bovine mastitis. The lack of comprehensive clinical metadata from the source herd also limited epidemiological associations. Future research should focus on expansive longitudinal surveillance to determine the prevalence and spread of ST3475 and related lineages. Multi-omics approaches and advanced *in vitro* models using bovine primary cells could further elucidate precise molecular mechanisms of host adaptation and pathogenicity.

## Conclusions

Based on a nationwide epidemiological survey of bovine mastitis in China from 2021 to 2024, this study reports the first isolation and genomic characterization of a novel *A. baumannii* sequence type, ST3475, from milk samples within that cohort. Through comparative genomic, phenotypic, and pathogenicity analyses with the previously known ST3256 strain, we systematically characterized the virulence and resistance profile of these two isolates. The ST3475 genome features a unique complement of phage elements, carbohydrate-active enzymes, and the carbapenemase gene *bla*OXA-344, with functional annotations revealing enrichment in infection-related pathways such as signal transduction and membrane transport. Phenotypically, the ST3475 isolate demonstrated enhanced growth kinetics, biofilm formation, adhesion and invasion of bovine mammary epithelial cells, and elevated pro-inflammatory cytokine induction relative to ST3256, under the conditions tested. *In vivo* models confirmed its heightened pathogenicity in this comparison, with significantly greater lethality in *G. mellonella* and more severe mammary tissue damage and inflammation in mice. Whereas both isolates exhibited multidrug resistance to β-lactams, ST3475 additionally carried the distinctive *bla*OXA-344 carbapenemase gene. Phylogenetically affiliated with the animal-associated CC2 clonal complex, ST3475 represented a newly identified sequence type with virulence and resistance characteristics in the isolates studied. These findings underscored the role of livestock as potential reservoirs for emerging resistant pathogens and highlighted the One Health imperative for integrated surveillance, prudent antimicrobial use in agriculture, and cross-sectoral collaboration to mitigate zoonotic transmission risks. This study provided insights that may inform targeted control of bovine mastitis and contribute to public health risk assessment related to *A. baumannii*, although broader lineage-level conclusions will require detailed investigations of additional isolates.

## Data Availability

The datasets presented in this study can be found in online repositories. The names of the repository/repositories and accession number(s) can be found below: https://www.ncbi.nlm.nih.gov/, ST3475: JBSLMF000000000.
